# A Simplified, Low-Cost Method for Polarized Light Microscopy

**DOI:** 10.4269/ajtmh.2009.09-0383

**Published:** 2009-11

**Authors:** Richard J. Maude, Wanchana Buapetch, Kamolrat Silamut

**Affiliations:** Centre for Tropical Diseases, Nuffield Department of Clinical Medicine, John Radcliffe Hospital, Oxford, United Kingdom; Mahidol-Oxford Tropical Medicine Research Unit, Faculty of Tropical Medicine, Mahidol University, Bangkok, Thailand

## Abstract

Malaria pigment is an intracellular inclusion body that appears in blood and tissue specimens on microscopic examination and can help in establishing the diagnosis of malaria. In simple light microscopy, it can be difficult to discern from cellular background and artifacts. It has long been known that if polarized light microscopy is used, malaria pigment can be much easier to distinguish. However, this technique is rarely used because of the need for a relatively costly polarization microscope. We describe a simple and economical technique to convert any standard light microscope suitable for examination of malaria films into a polarization microscope.

Hemozoin is a byproduct of heme metabolism by malaria parasites. In blood films and histologic specimens, it can often be seen as an intracellular inclusion body in parasitized erythrocytes and leukocytes. It also can be extracellular when released by cell rupture during slide preparation. This inclusion body can vary in color from black to golden brown and is most common in association with late-stage parasites or as an indicator of previous (including treated) infection. Because it is a birefringent (doubly refracting) substance, it is significantly more visible when viewed using crossed polarized light.^1^ This viewing is possible if one uses a polarizing microscope that uses two polarizing filters at right angles to each other, one between the light source and the slide and the other between the slide and the observer.

In one study examining histologic specimens, the sensitivity of polarizing microscopy for malaria parasites was almost double that of conventional light microscopy although 1 of 20 infections detected by polarizing microscopy were false-positives results.^2^ These false-positive positive results occur because dust and dirt on the slide can sometimes produce a birefringence similar to that of hemozoin, although this can usually be avoided by double-checking the pigment-containing cells for parasites by using light microscopy. Polarizing microscopy has also been used to quantify past episodes of placental malaria in placental blood smears, and a related method using a depolarized laser has been tested for automated detection of malaria in peripheral blood by flow cytometry.^3^,^4^

There are several commercially available polarizing microscopes currently available, typically priced at approximately $1,000–$7,000 U.S. dollars. We have devised a simple method for adapting a conventional light microscope for polarized light microscopy for as little as $10 U.S. dollars. Although the result does not match the high-precision optics of a purpose-built instrument, we found it to be perfectly adequate for detecting malaria pigment.

The minimum equipment required for this technique is a microscope suitable for examination of malaria blood films, a small piece of cardboard, a pair of gray or black polarizing sunglasses (the analyzer), and a small additional piece of polarizing material (the polarizer), e.g., a 1.5 cm × 1.5 cm piece cut out of a plastic polarizing lens or a polarizing test strip commonly provided free with polarizing sunglasses.

The technique is as follows. First, look through the sunglasses and the analyzer, with one eye, at a distant object. Rotate the analyzer in a clockwise direction until the object appears as dark as possible. You will notice if you rotate the analyzer further that the object will become light again. The difference between light and dark will be about 45° of rotation. Note the angle at which the object appears darkest. The analyzer should then be mounted at this angle in the piece of cardboard by folding the cardboard in half, cutting a 1 cm × 1 cm round hole through both layers in the center, and taping the analyzer in place between the two layers, covering the holes. Second, lower the condenser on the microscope and place the mounted analyzer over the light source. Raise the condenser to hold the analyzer in place. Third, place the microscope slide to be examined on the platform and switch on the microscope light source, turning the brightness to maximum. The user should then examine the slide as normal but while wearing the polarizing sunglasses. The slide will appear darker and more red than usual and malaria pigment will stand out from the background as a white spot, often with a hint of red or brown round the edges. By turning the head 45° to the right or left, the user will notice the color of the pigment becomes darker and the transparency is lost. This is caused by birefringence of the malaria pigment. Using the method described previously,^5^ with the camera lens covered by the sunglasses, one can take photographs through the microscope (Figure 1). Alternatively, an additional piece of polarizing material large enough to cover the microscope eyepiece can be held between the microscope eyepiece and the eye or camera lens in place of the sunglasses and rotated as described above.

**Figure 1.**
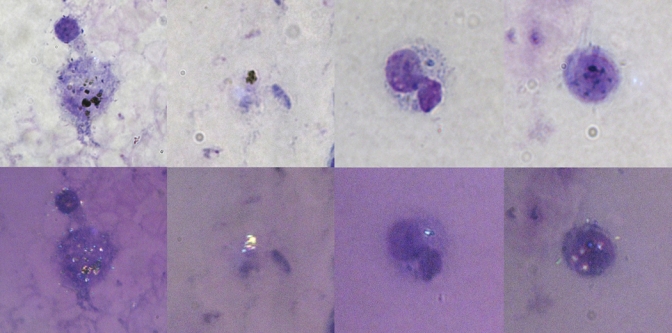
*Plasmodium falciparum* malaria pigment on a thick blood film from a patient with severe malaria. The upper panel shows pale images taken by using conventional light microscopy, and lower panel shows dark images taken by using the polarized light method described. This figure appears in color at www.ajtmh.org.
